# Influence of Preventive Tooth Extractions on Quality of Life in Patients with Antiresorptive Intake—A Prospective Longitudinal Study

**DOI:** 10.3390/ijerph182111650

**Published:** 2021-11-06

**Authors:** Thomas Rückschloß, Julius Moratin, Sven Zittel, Maximilian Pilz, Christoph Roser, Michael Engel, Christian Freudlsperger, Jürgen Hoffmann, Oliver Ristow

**Affiliations:** 1 Department of Oral and Maxillofacial Surgery, University of Heidelberg, Im Neuenheimer Feld 400, D-69120 Heidelberg, Germany; julius.moratin@med.uni-heidelberg.de (J.M.); sven.zittel@med.uni-heidelberg.de (S.Z.); micheal.engel@med.uni-heidelberg.de (M.E.); christian.freudlsperger@med.uni-heidelberg.de (C.F.); juergen.hoffmann@med.uni-heidelberg.de (J.H.); oliver.ristow@med.uni-heidelberg.de (O.R.); 2 Department of Biometry, Institute of Medical Biometry and Informatics, University of Heidelberg, Im Neuenheimer Feld 130.3, D-69120 Heidelberg, Germany; pilz@imbi.uni-heidelberg.de; 3 Department of Orthodontics, University of Heidelberg, Im Neuenheimer Feld 400, D-69120 Heidelberg, Germany; christoph.roser@med.uni-heidelberg.de

**Keywords:** quality of life, tooth extraction, bisphosphonates, denosumab, EQ-5D

## Abstract

Background: To find out whether preventive tooth extractions in patients on antiresorptive therapy have a direct impact on the patients’ overall quality of life (QoL); Methods: QoL using the five-level version of the EuroQol Group’s EQ-5D instrument (EQ-5D-5L) was longitudinally assessed in N = 114 prospectively enrolled patients with indication of preventive tooth extraction over a period of 12 months. Patients were stratified as high-risk (malignant disease with bone metastasis or multiple myeloma, with monthly high-dose antiresorptive therapy delivered intravenously [bisphosphonate] or subcutaneously [denosumab]) and low-risk/osteoporosis patients (weekly low-dose antiresorptive therapy administered orally [bisphosphonate] or half-yearly subcutaneously [denosumab]). The measurement time points were 4 weeks preoperatively (T0), 2 months (T1) and 1 year postoperatively (T2), respectively. Results: EQ-5D-5L index scores fell in a range from −0.21 to 1.00 in the low-risk group to 0.15 to 1.00 in the high-risk group. The *t*-test comparing the baseline index scores of both groups showed EQ-5D-5L index score in the low-risk group (0.708 ± 0.292) to be significantly smaller (*p* = 0.037) than in the high-risk group (0.807 ± 0.19). ANCOVA showed no significant differences in EQ-5D-5L index scores between the groups at T1 and T2. Conclusions: Preventive tooth extractions in patients undergoing antiresorptive treatment have no negative effect on QoL. Therefore, if indicated, preventive tooth extraction should not be omitted. Patient-oriented outcome measures are important to obtain a good risk–benefit balance for patient-specific treatment.

## 1. Introduction

Antiresorptive agents, in particular bisphosphonates and denosumab, are essential components of pharmacological therapy for avoiding skeletal complications in osteoporosis and malignant primary disease. These agents have beneficial effects on health-related quality of life (QoL) [1]. In essence, antiresorptive agents are very well tolerated and their use is considered to have few adverse reactions. However, in past years, the medication-related osteonecrosis of the jaw (MRONJ) has been shown to be a significant side effect of antiresorptives [2,3,4]. MRONJ can lead to a complication-laden course with the loss of dental and oral function, with possible large-volume loss of jaw sections and thereby a reduction in QoL [5,6]. Furthermore, despite the lack of evidence, the diagnosis of MRONJ and sometimes even the suspicion of diagnosis leads to an interruption of antiresorptive and oncologic therapy. This again may have an impact on the underlying disease [7] and indirectly on the QoL.

Therefore, it is of utmost importance to prevent the development of MRONJ. The pathogenesis of MRONJ has not been definitively elucidated at this time. However, current data indicate that a variety of cellular and molecular mechanisms are involved in the development of MRONJ, and the reduced vascularization observed in BPs-treated patients has been suggested as the key player for the necrosis of the jaw [8]. However, infection-associated lesions often precede the development of necrosis of the jaw. Consequently, events occurring during the establishment of the infection result as the trigger for the exacerbation of MRONJ. For a long time, it was believed that tooth extractions were the main trigger for MRONJ. As a consequence, many guidelines still recommend avoiding tooth extractions under antiresorptive therapy [9,10,11]. However, it is becoming increasingly clear that the triggering factor is not the tooth extraction itself, but the local infection leading to or following tooth extractions [12,13]. Data in recent years clearly show that tooth extractions under antiresorptive therapy can be performed safely if certain rules are followed [14,15]. Furthermore, there are a plethora of studies that suggest high rates of necrotic changes to the alveolar bone structure at the time of tooth extraction. With this knowledge one must assume that excessive delay to extracting an inflammatory tooth increases the risk of MRONJ development more than the extraction itself if the well-established preventive measures are met [16]. Therefore, international guidelines have adapted their recommendations [3,17,18,19]. However, to date, there are no data which evaluate the impact of preventive tooth extractions on patients’ QoL. Especially but not exclusively in oncological patients with a palliative overall prognosis, QoL is an important patient-oriented influencing factor [20]. Therefore, it is also important to include the QoL aspect in the weighing of risks and the treatment decision of a preventive tooth extraction.

The aim of this study was to find out to what extent patients’ general QoL is affected by preventive tooth extraction in osteoporosis and tumor patients and whether there were differences between high (tumor) and low risk (osteoporosis) patients.

## 2. Materials and Methods

### 2.1. Study Design

As an extension analysis of the PRISM trial (Preventive Strategies in Medication-Related Osteonecrosis of the Jaw), this study focuses on the secondary outcome QoL. PRISM is a prospective, parallel-group, open-label, randomized clinical pilot trial which compares different wound closure techniques, specifically the sub-periosteal prepared muco-periosteal flap and the epi-periosteal prepared mucosa flap as well as the feasibility of alveoplasty after surgical tooth extractions in patients undergoing and after antiresorptive treatment [14]. The investigation was carried out at the Department of Cranio-, Oral-, and Maxillofacial Surgery, University of Heidelberg. The study protocol was approved by the designated Research Ethics Board (Ethics number S-088/2016), registered in the German Clinical Trials Register (DRKS00010106), and conducted with the understanding and written consent of each patient in full accordance with ethical principles and the Declaration of Helsinki in its current version.

### 2.2. Patients

All patients who underwent therapeutic antiresorptive therapy with bisphosphonates or denosumab and who have been assigned to our department for teeth removal under preventive conditions were consecutively recruited and checked for eligibility in our specialized consultation hour over a period of 24 months between 2016 and 2018. The inclusion criteria for the PRISM trial were as follows: (a) indication of need for tooth removal; (b) ongoing antiresorptive treatment with bisphosphonates or denosumab; (c) no signs of exposed bone to the oral cavity, and therefore no signs of MRONJ [9]. The exclusion criteria were as follows: (a) history of head and neck radiation, (b) metastatic bone disease of the maxillofacial region; (c) patients younger than 18; and (d) incomplete data sets.

For our secondary extension analysis, we chose only patients whose treatment was performed under drug holiday (antiresorptive treatment was paused 4 weeks before surgery and continued 4 weeks after surgery) and follow up of 1 year after surgery was completed. Furthermore, we stratified patients either as tumor patients (high risk patients; malignant disease with bone metastasis or multiple myeloma, with monthly high-dose antiresorptive therapy delivered intravenously [bisphosphonate] or subcutaneously [denosumab]) or as osteoporosis patients (low risk patients, weekly low-dose antiresorptive therapy administered orally [bisphosphonate] or half-yearly subcutaneously [denosumab]).

### 2.3. Data Collection

To minimize performance bias, all dental extractions were performed under local anesthesia by the same surgeons who were experienced in preventive tooth extractions during/after antiresorptive treatment. Prior to initiation, all involved surgeons were trained in the techniques. All surgeons followed the surgical protocol as published previously [14], in adherence to a standardized intra-institutional protocol following the German guidelines for MRONJ [18]. After the operation, further follow-up care was provided in our weekly consultation hours.

Four weeks preoperatively (T0), 2 months (T1) and 1 year postoperatively (T2) all patients filled out the five-level version of the EuroQol Group’s EQ-5D instrument (EQ-5D-5L). This questionnaire is used to assess health-related quality of life. It measures patients’ health status in five dimensions (mobility, self-care, capacity to undertake usual activities, pain and discomfort, and anxiety and depression) which each have five levels of severity (no problems, slight problems, moderate problems, severe problems and either extreme problems or unable to perform activity) [21]. Patients described their health status by selecting one statement in each dimension, ranging from 1 (no problem) to 5 (extremely problematic). The EQ-5D-5L further offers the opportunity to calculate an overall score, the so-called single index utility score, from the five answers of a patient. To convert an individual EQ-5D-5L health state to single index utility score a value set is required. The value sets are based on the results of valuation studies for different countries/regions. A value set is essentially a set of weights for each of the levels in the five EQ-5D dimensions. A large weight means that people in that country/region believe that a particular level of severity has a large impact on health-related quality of life. In this trial, responses to EQ-5D-5L questionnaires were converted into a single index utility score using the German value set [22], resulting in values from 1 (perfect health) to 0 (death). The EQ-5D-5L included the EQ-visual analog scale (EQ-VAS) which respondents used to rate their overall health. The top and bottom endpoints are “the worst health you can imagine” (0) and “the best health you can imagine” (100).

### 2.4. Statistical Analysis

All the statistical computations were carried out using the statistical software R version 4.0.2. Because of the preliminary “proof-of-concept” character of the PRISM trial, no formal sample size calculation was performed. Descriptive statistics were used to characterize patient demographics and clinical details. Continuous variables were reported in terms of mean values and categorical variables as numbers and percentages. The sample data were described by frequencies (%) or mean for EQ-5D score. Variability around mean values was measured by standard deviations (SD). The presentation of the EQ-5D-5L results followed the recommendations of the EuroQol Group [21]. To compare quality of life as presented by EQ-5D-5L index score and EQ-VAS and between high and low-risk groups, an ANCOVA model that uses the respective post-intervention value as outcome was computed, together with the baseline-value and the treatment group, as recommended by the current literature. The baseline-values were compared using a standard *t*-test. Only patients with complete data on all measured variables were included.

## 3. Results

### 3.1. Patient Characteristics

160 patients participated in the PRISM trial, while 132 completed the 1-year follow-up. Out of these 132 patients, 114 fulfilled the inclusion criteria and were included (85 females and 29 male patients). The data sets of all patients were complete. 54 were stratified as osteoporosis/low risk patients and 60 as tumor patients/high risk patients. Patients’ underlying diseases/cancer types and antiresorptive treatment prior to extraction are shown in Table 1. None of the patients included developed MRONJ or any skeletal-related event until T2.

### 3.2. Five-Level Version of the EuroQol Group’s EQ-5D Instrument (EQ-5D-5L)

Please refer to Table 2 and Table 3 for patients’ responses. EQ-5D-5L index scores fell in the range from −0.21 to 1.00 in the low-risk group to 0.15 to 1.00 in the high-risk group. The *t*-test which compared the baseline index scores of both groups shows that EQ-5D-5L index score in the low-risk group (0.708 ± 0.292) is significantly smaller (*p* = 0.037) than in the high-risk group (0.807 ± 0.19). This means that health-related QoL at the baseline of patients in the osteoporosis group was significantly worse than that in the tumor group. ANCOVA showed no significant differences in EQ-5D-5L index scores between the groups at T1 and T2 (Table 4).

EQ-VAS fell in the range from 5 to 100 in the low-risk group and 10 to 1000 in the high-risk group. The t-test comparing the EQ-VAS values of both groups shows no significant (*p* = 0.146) differences between the low-risk (61.76 ± 25.29) and high-risk groups (68.00 ± 19.49). Also, ANCOVA showed no significant differences in EQ-5D-5L index scores between the groups at T1 and T2 (Table 4).

## 4. Discussion

Many authors and professional societies are still of the opinion that tooth removal is the main trigger for necrosis [9,10,11]. According to this view, MRONJ following dental extraction could seriously impair patients’ quality of life and delay oncological therapy [23]. In a number of studies, however, it has been shown that tooth extractions carried out under appropriate preventive provisos (antibiotic therapy, atraumatic surgery, alveoplasty, and primary wound closure) only rarely result in MRONJ [14,15]. Nonetheless, it still cannot be assumed that the prevention of MRONJ leads to a positive influence on the quality of life. In addition, there are no data to date concerning the impact of preventive tooth extraction on QoL itself. However, this patient-oriented outcome measure is important to obtain a good risk–benefit balance for patient-specific treatment planning.

To the best of our knowledge this study is the first to evaluate the influence of preventive tooth extraction on patients’ QoL. The results of this study show that there is no significant change of QoL between the baseline score and after surgery, either for the osteoporosis (EQ-5D-5L index scores T0 0.71 ± 0.29; T1 0.77 ± 0.26) or the tumor group (T0 0.81 ± 0.19; T1 0.80 ± 0.23). The same applies for the scores 12 months after surgery.

Overall, data on QoL in MRONJ patients are lacking. A recent systematic review on this topic identified only eight studies that investigated quality of life of patients suffering from MRONJ [23]. Five of the studies looked at oral health-related QoL (using the questionnaires: OHIP 14, QLQ-HN35) [24,25,26,27,28] while seven looked at general health-related QoL (using the questionnaires: EORTC QLQ-C30, SF 12, UWQoL, EQ-5D) [20,24,25,27,28,29,30]. The studies examined the effects of MRONJ alone as well as that of different therapy methods on patients’ QoL. It was shown that patients suffering from MRONJ generally have a poor quality of life and several oral complaints, including pain and speech problems [23]. A higher stage of disease seemed to result in worse QoL [24]. However, it should be noted that the underlying disease makes it difficult to interpret these results accurately. In fact, it is difficult to distinguish whether the reduction in quality of life is caused by the underlying disease or a MRONJ [23]. When comparing initial QoL between patients with osteoporosis or cancer, Oteri et al. found that differences exist. Therefore, it is recommended to measure oral QoL, as it focuses on the disease process and reduces interference from other comorbidities [27].

In this study, we deliberately chose not to assess oral QoL but QoL as a whole. The first reason for this was that it seems clear that the indication for tooth extraction and the postoperative situation after extraction will most certainly influence oral QoL, so the statement is difficult to evaluate and does not make sense in the case of a clearly given indication. Secondly, since the major aim of this study was to find out in a differentiated way whether the preventive tooth extraction has an influence on patients’ general condition, we thought it to best include this patient-oriented factor in a risk-benefit assessment

Another fear of many health practitioners is that a drug holiday of antiresorptive treatment could lead to a progression of the underlying disease, skeletal-related events such as pathologic fracture, radiation or surgery to the bone and spinal cord compression. These are all associated with high mortality [31] and their functional burden prolongs years after the event [32], placing a significant burden on health care resources [33].

In the present study, a drug holiday has not caused any deterioration of QoL in our collective. Nor does the QoL of osteoporosis and tumor patients differ significantly. However, it has to be mentioned that in the present study only a drug holiday of 2 months was performed. It is questionable whether this short drug interruption has any influence at all on the pharmacokinetics, especially for bisphophonates concerning their physical binding to the bone and the associated half-life of many years. Although there are increasing hints that a drug holiday has no influence on outcome after tooth extractions, high-level evidence is still lacking (15). Based on these data, the updated German guidelines and the statement of the European task force also make a very cautious recommendation for a drug holiday [3,18]. It is additionally important to mention that in all cases the drug holiday had previously been initialized by the attending oncologist or osteologist prescribing the antiresorptive and, therefore, we were only marginally involved in the decision.

The main limitation of this study is that only a questionnaire was used, which depicted the overall health-related QoL of the patients and did not refer to oral health. Furthermore, the heterogeneous intake of antiresorptive medication might be considered as a limitation to the study protocol. The small number of cases is also critical. For these reasons, the application of multifactorial regression models (considering covariants as a medication type) is not possible. Furthermore, for future studies it would be interesting to have more details on the indications leading to extraction. For the pre- and postoperative “drug holiday,” we followed our unit-internal protocol. Interestingly, in almost all cases presented, the drug holiday had previously been initialized by the attending oncologist or osteologist prescribing the antiresorptive and, therefore, we were only marginally involved in the decision. Although hints are increasing that a drug holiday has no influence on outcome, high-level evidence is still lacking. Future studies, therefore, ought to consider both oral and overall health and include more patients to enable the use of multifactorial regression models.

## 5. Conclusions

In conclusion, preventive tooth extractions in patients under antiresorptive treatment have no negative effects on the QoL. Therefore, if indicated, preventive tooth extraction should not be omitted. Patient-oriented outcome measures are important to obtain a good risk–benefit balance for patient-specific treatment planning in patients who undergo antiresorptive treatment.

## Figures and Tables

**Table 1:** Descriptive statistics.

	Osteoporosis		Tumor	
	*n*	%	*n*	%
Gender				
Female	45	83.3	40	66.7
Male	9	16.7	20	33.3
Age [years]				
Mean	70.2		67.8	
SD	9.5		11.5	
Cancer types				
Breast			32	53.3
Prostate			6	10.0
Renal			2	3.3
Multiple myeloma			16	26.7
Others			4	6.8
Bone metastasis				
No	54	100.0	17	28.3
Yes			27	45.0
Multiple Myeloma			16	26.7
Antiresorptive Treatment				
Bisphosphonates	43	79.6	38	63.3
Zoledronic acid	9	18.4	36	60.0
Ibandronic acid	2	4.1	2	3.3
Alendronic acid	27	55.1		
Risedronic acid	3	6.1		
Others	2	4		
Denosumab	6	11.1	17	28.3
Both	5	9.3	5	8.3
Duration antiresorptive treatment [months]				
Mean	49.8		43.8	
SD	44.7		42.4	

**Table 2:** Results of EuroQol Group’s EQ-5D instrument (EQ-5D) for high-risk group.

	High-Risk Group					
	T0		T1 (4 Weeks)		T2 (1 Year)	
	*n*	%	*n*	%	*n*	%
**Mobility**						
No problems	35	58.3	34	56.7	41	68.3
Slight problems	9	15.0	9	15.0	4	6.7
Moderate problems	13	21.7	11	18.3	8	13.3
Severe problems	3	5.0	4	6.7	6	10.0
Unable to walk about	0	0	2	3.3	1	1.7
**Self-care**						
No problems	44	73.3	45	75.0	44	73.3
Slight problems	8	13.3	7	11.7	9	15.0
Moderate problems	5	8.3	5	8.3	3	5.0
Severe problems	1	1.7	2	3.3	3	5.0
Unable to to wash or dress	2	3.3	1	1.7	1	1.7
**Usual activities**						
No problems	35	58.3	30	50.0	36	60.0
Slight problems	11	18.3	15	25.0	9	15.0
Moderate problems	10	16.7	8	13.3	9	15.0
Severe problems	3	5.0	7	11.7	6	10.0
Unable to do usual activities	1	1.7	0	0	0	0
**Pain/discomfort**						
No pain/discomfort	18	30.0	18	30.0	20	33.3
Slight pain/discomfort	12	20.0	18	30.0	17	28.3
Moderate pain/discomfort	20	33.3	17	28.3	14	23.3
Severe pain/discomfort	10	16.7	6	10.0	9	15.0
Extreme pain/discomfort	0	0	1	1.7	0	0
**Anxiety/depression**						
Not anxious/depressed	29	48.3	34	56.7	38	63.3
Slightly anxious/depressed	14	23.3	15	25.0	13	21.7
Moderately anxious/depressed	14	23.3	8	13.3	6	10.0
Severely anxious/depressed	3	5.0	3	5.0	3	5.0
Extremely anxious/depressed	0	0	0	0	0	0

**Table 3:** Results of EQ-5D for low-risk group.

	Low-Risk Group					
	T0		T1 (4 Weeks)		T2 (1 Year)	
	*n*	%	*n*	*%*	*n*	*%*
**Mobility**						
No problems	26	48.1	32	59.3	24	44.4
Slight problems	8	14.8	7	13.0	10	18.5
Moderate problems	12	22.2	7	13.0	11	20.4
Severe problems	6	11.1	4	7.4	7	13.0
Unable to walk about	2	3.7	4	7.4	2	3.7
**Self-care**						
No problems	38	70.4	44	81.5	39	72.2
Slight problems	4	7.4	4	7.4	7	13.0
Moderate problems	7	13.0	3	5.6	3	5.6
Severe problems	4	7.4	2	3.7	4	7.4
Unable to to wash or dress	1	1.9	1	1.9	1	1.9
**Usual activities**						
No problems	29	53.7	28	51.9	27	50.0
Slight problems	5	9.3	9	16.7	9	16.7
Moderate problems	11	20.4	7	13.0	9	16.7
Severe problems	7	13.0	8	14.8	7	13.0
Unable to do usual activities	2	3.7	2	3.7	2	3.7
**Pain/discomfort**						
No pain/discomfort	12	22.2	13	24.1	11	20.4
Slight pain/discomfort	9	16.7	17	31.5	14	25.9
Moderate pain/discomfort	16	29.6	14	25.9	17	31.5
Severe pain/discomfort	12	22.2	9	16.7	10	18.5
Extreme pain/discomfort	5	9.3	1	1.9	2	3.7
**Anxiety/depression**						
Not anxious/depressed	23	42.6	27	50.0	29	53.7
Slightly anxious/depressed	14	25.9	19	35.2	10	18.5
Moderately anxious/depressed	13	24.1	5	9.3	9	16.7
Severely anxious/depressed	2	3.7	3	5.6	3	5.6
Extremely anxious/depressed	2	3.7	0	0	3	5.6

**Table 4:** Comparison of the high-risk group and the low-risk group over the time points T0, T1, and T2.

	Time Point	High-Risk Group		Low-Risk Group		*p*-Value for Group Difference
		Mean	SD	Mean	SD	
Index score	T0	0.81	0.19	0.71	0.29	0.037 *
	T1	0.80	0.23	0.77	0.26	0.321
	T2	0.82	0.22	0.74	0.27	0.691
EQ-VAS	T0	68.00	19.49	61.76	25.29	0.146
	T1	69.17	21.75	69.06	24.38	0.304
	T2	69.13	22.97	67.37	24.36	0.540

* *p* < 0.05.

## Data Availability

The data presented in this study are available in justified cases on request from the corresponding author.
